# Validation and reliability of a prototype orthodontic bracket debonding device equipped with force-sensitive resistor (FSR): a novel method of measuring orthodontic bracket debonding force in vivo

**DOI:** 10.1186/s40510-019-0277-x

**Published:** 2019-07-08

**Authors:** Tamzid Ahmed, Norma Ab Rahman, Mohammad Khursheed Alam

**Affiliations:** 1 Department of Science of Dental Materials, Bangladesh Dental College, Dhaka, Bangladesh; 2 Orthodontic Unit, School of Dental Sciences, Universiti Sains Malaysia, Kota Bharu, Kelantan Malaysia; 3 Department of Orthodontics, College of Dentistry, Jouf University, Sakaka, Kingdom of Saudi Arabia

**Keywords:** Orthodontic bracket, In vivo, Debonding force, Prototype device

## Abstract

**Background:**

To introduce an orthodontic bracket debonding device capable of measuring debonding force clinically by a novel sensor mechanism

**Materials and method:**

A prototype orthodontic debonding device was constructed utilizing a lift-off debonding instrument (LODI) and force-sensitive resistor (FSR). For data interpretation, the force sensor was equipped with a microcontroller and C++ programming software running on a computer. Ninety-nine (99) 0.022-in. conventional metallic brackets were bonded to premolar teeth in vitro by a single clinician applying the same adhesive and bonding technique. For validation, the mean debonding force measured by the prototype debonding device (*n* = 30) and the universal testing machine (*n* = 30) was compared. Both intra- and inter-examiner reliability tests were done by holding and operating the device in a standardized manner. Following debonding by the prototype device, the bracket failure pattern was evaluated (*n* = 30) by adhesive remnant index (ARI) under the stereomicroscope at × 30 magnification. Statistical analysis included independent samples *t* test for validation and intraclass correlation coefficient (ICC) with a 95% confidence interval for both intra- and inter-examiner reliability.

**Results:**

Mean orthodontic bracket debonding force measured by the prototype device (9.36 ± 1.65 N) and the universal testing machine (10.43 ± 2.71 N) was not significantly different (*p* < 0.05). The prototype device exhibited excellent intra- [ICC (3, 1) = 0.942] and inter-examiner reliability [ICC (2, 1) = 0.921] and was able to debond brackets mostly at the bracket-adhesive interface.

**Limitation:**

Due to adjusting the position and mechanism of the force sensor, the device had to be held in a modified standardized position.

**Conclusion:**

A novel method of measuring in vivo orthodontic bracket debonding force has been introduced which proved to be validated, reliable, and safe in terms of enamel damage.

## Introduction

Brackets are one of the major structural components of the fixed orthodontic appliances, responsible for the biomechanical tooth movement in the desired direction in order to correct the malocclusion. Therefore, the success of the treatment relies much on the stable bonding between the brackets and the tooth surface on which they are attached by means of an orthodontic adhesive. Researchers and manufacturers introduced various brackets, adhesives, surface preparation techniques, and bonding methods to reduce the clinical bracket failure rate. Laboratory-based in vitro or ex vivo mechanical tests are usually done to evaluate their bonding efficiency. Either true shear or tensile force is applied by the universal testing machine until debonding. The average stress is calculated and then interpreted as the bond strength of the test specimen. By such experiments, certain physical and chemical properties favorable to adhesion can be explained but the actual performance of a material should be tested in the environment where it is expected to function [1]. Biodegradation or aging of the dental material is a common phenomenon due to prolonged exposure to the variations of temperature and pH, saliva, bacterial byproducts, combined forces of mastication, and activated archwires inside oral environment [2, 3]. Biodegradation has a negative impact on the adhesion properties of the orthodontic dental materials as different studies reported to have the lower bond strength of orthodontic brackets in vivo in comparison to in vitro [4–6].

Although universal testing machine is considered the “gold standard” in terms of accuracy and precision, but some drawbacks were noted when replicating clinical bracket debonding: they can apply either true shear force or tensile force, but clinically, brackets are exposed to combined shear, tension, and torsional loading modes during function and as well as during clinical debonding [7]; they apply force at much lower impact velocity than in clinical situations of debonding [1]; and last of all, due to the large dimensions, they cannot be introduced clinically. Hence, it was emphasized on the introduction of a device that is designed to debond brackets clinically according to the manufacturer’s direction while providing the quantitative magnitude of the applied force [1]. Various prototypes were introduced equipped with either digital force gauge (DFG) or strain gauge [4, 6, 8–13]. They were attached either with the modified elastic spacer instrument or the manufacturer-made debonding pliers. Prototypes with DFG and modified elastic spacer instrument are complex as acrylic splints are required to be worn by the subjects to prevent enamel damage during debonding [4, 6, 9, 11]. On the other hand, strain gauge relies on the deformation of the plier handles on which it is attached. Hence, the results may vary with the plier types and the manner in which they are held [13].

Therefore, the present study introduced a prototype orthodontic bracket debonding device equipped with a force-sensitive resistor (FSR). Unlike strain gauges, FSR relies on the direct force application. They are also thin, inexpensive, dynamic, durable, and easy to install. Before applying clinically, the study aimed to validate the prototype by comparing with the universal testing machine and also test the intra- and inter-examiner reliability.

## Materials and method

### Development and mechanism of the prototype device

**Fig. 1:**
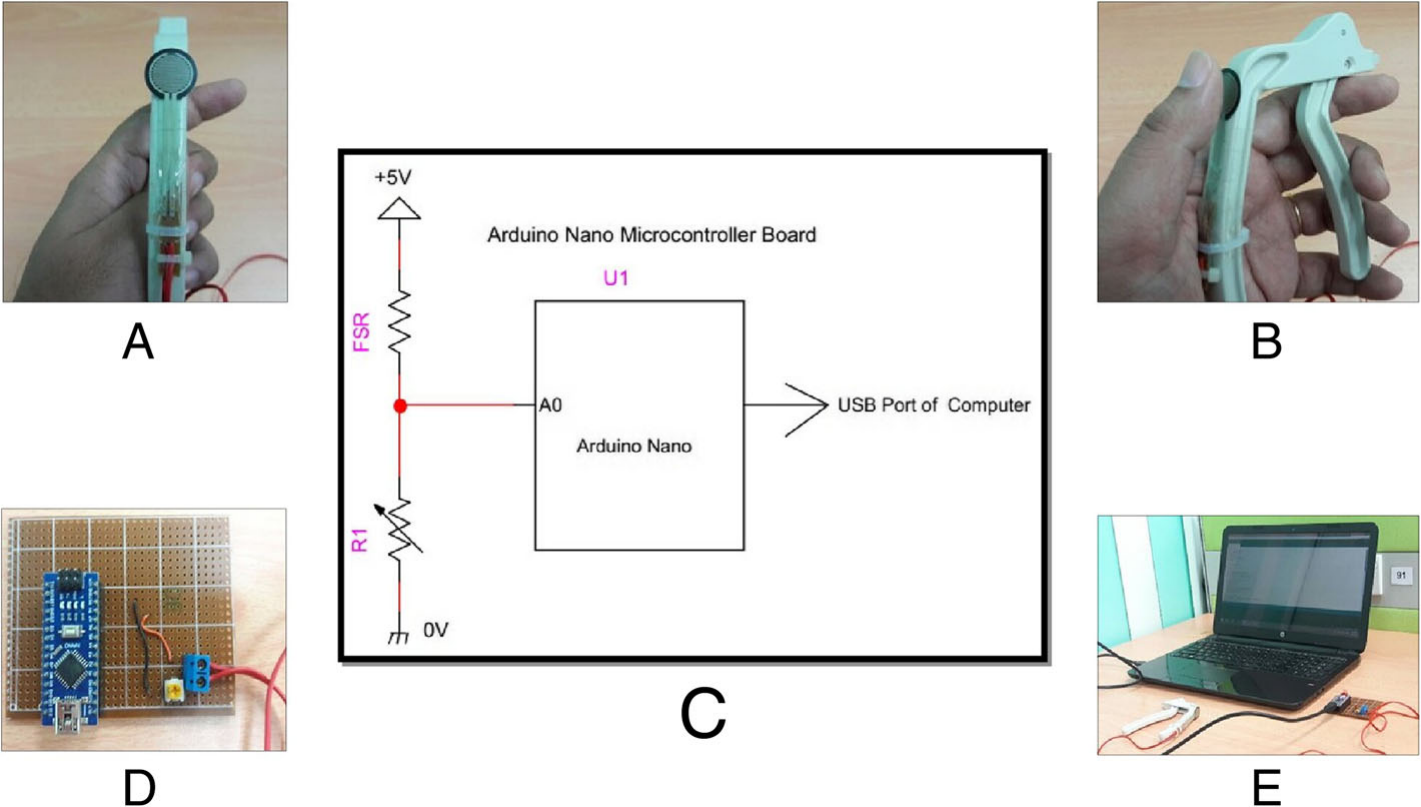
Mechanism of the prototype device. **a** Attachment of FSR to LODI. **b** Griping position of the device. **c** Circuit diagram. **d** Microcontroller. **e** C++ programming software

The FSR is an electric sensor made of thick polymer film that changes its resistance or conductance value when force or pressure is applied [14]. The electrical resistance decreases with the increase of force. In the present study, FSR (Model: 402, Interlink, CA, USA) was attached to a lift-off debonding instrument (LODI) (3M Unitec, Monrovia, CA, USA) to measure the orthodontic bracket debonding force (Fig. 1a). The FSR was attached on the posterior arm of the LODI by an adhesive tape and cable tie. The FSR is nominally 0.46 mm thick with a circular force-sensitive area of 12.5 mm diameter at the top. The holding position of the prototype device is standardized by keeping the thumb on the active area of the force sensor and the rest of the fingers on the anterior arm (Fig. 1b).

To measure the resistance on the FSR, a voltage divider circuit was constructed with two series resistor where one resistor is FSR (Fig. 1c). Then, the following formula was applied to measure the resistance of the FSR:

$$ {\displaystyle \begin{array}{c}\ \mathrm{FSR}=\frac{R\times \left(V-{V}_R\right)}{V_R}\\ {}{V}_R-\mathrm{output}\ \mathrm{voltage},V-\mathrm{input}\ \mathrm{voltage},R-\mathrm{resistor}\\ {}\mathrm{Here},V=5\ \mathrm{V}\ \mathrm{and}\ R=6.06\ \mathrm{K}\Omega .\end{array}} $$

The output voltage across the series resistor was measured by a simple C++ programming language running on an analog to digital converter (ADC) module of an Atmega 328 microcontroller (Shenzhen Rhino Technology Co., Ltd., Shenzhen, Guangdong, China) with in-built USB (universal serial bus) (Fig. 1d). The C++ programming was designed to select the maximum output voltage value across the resistor which was then applied to the abovementioned formula to determine the resistance of the FSR. The formula was also computed by the C++ programming.

### Calibration of the force sensor

Before installing the FSR on the plier handle, calibration was done. A calibration weight scale was used, and the known weights of 20, 50, 100, 200, and 500 g were placed sequentially on the force sensor each for 10 s. The sensor outputs were recorded in resistance. The resistance values of FSR against these known force values were then converted into conductance (1/resistance). A linear calibration curve (*R*^2^ = 0.99, *p* < 0.001) was obtained, and unknown force values were calculated from the following regression equation:

$$ {\displaystyle \begin{array}{c}\mathrm{Force}\ \left(\mathrm{in}\ \mathrm{gram}\hbox{-} \mathrm{force}\ \mathrm{units}\right)\times 0.0098=-11.733\ \left(\mathrm{constant}\right)+\left(1461262.434\times \mathrm{conductance}\right)\\ {}\left(\mathrm{Note}:1\ \mathrm{gram}\hbox{-} \mathrm{force}=0.0098\ \mathrm{Newton}\right)\\ {}\mathrm{Force}\ \left(\mathrm{in}\ \mathrm{Newton}\ \mathrm{units}\right)==-11.733\ \left(\mathrm{constant}\right)+\left(1461262.434\times \mathrm{conductance}\right)\end{array}} $$

### Sample size calculation

All the calculations were done with 80% power and alpha error probability 0.05.

The sample size for the validation study was calculated by the G Power software, version 3.1 [15]. The input parameters were *t* test, tail(s) one, effect size *d* 0.65, allocation ratio N2/N1–1. Total sample size calculated was 60.

For the reliability study, the calculation was done using the Power Analysis and Sample Size (PASS) software (version 11.0.7, PASS, NCSS, LLC). The input parameters were ICC test; number of raters, *k*-2; null hypothesis, *R*_0_ = 0; and alternate hypothesis, *R*_1_ = 0.7. The calculated sample size was ten (10) per group which means a total of 30 sample size.

### Sample preparation

Ninety-nine (99) extracted human maxillary premolar samples were collected. Following extraction, the samples were cleansed of the calculus and periodontal ligaments and then stored in distilled water at room temperature. For the universal testing machine group, thirty (30) samples were prepared by inserting the tooth roots vertically and centrally into a metallic cylinder of 22-mm length and 8-mm diameter filled with acrylic resin (Interacryl Cold, Interdent, Slovenia). The inner surface of the cylinder was coated with petroleum jelly, and the samples were withdrawn after polymerization. For the prototype device, a silicon mold of 33 × 15 × 15 mm was used to prepare the sample holders in a similar manner.

A standardized bonding protocol was maintained by a single clinician. Prior bonding, tooth surface was prophylactically polished with non-fluoride slurry of pumice in a bristle brush attached to a slow speed handpiece for 10 s followed by rinsing and air drying for another 15 s and 10 s respectively. Surface preparation was done by Transbond Plus self-etching primer (SEP) (3M Unitek, Monrovia, CA, USA) for 15 s with gentle air blow for the uniform liquid dispersion. Conventional 0.022 metallic brackets (HKS 3, Ortho Classic, McMinnville, USA) were coated with Transbond XT adhesive (3M Unitek, Monrovia, CA, USA) on the base. The brackets were then attached and pressed against the enamel surface by the bracket holding forceps. The excess adhesive around the bracket periphery was removed with a right-angled probe, and the adhesive was light-cured with a LED light curing unit (model DB686, COXO, Guangdong, China) for 20 s at a distance of 3 mm from the tooth surface. The intensity of the light curing was 1200 mW/cm^2^ at a wavelength of 420–480 nm. In all instances, the light curing tip was held for 10 s each at 45° angulation from the occlusal and gingival directions respectively [10]. All the specimen were bench cured for 10 min before storing them in the distilled water at the room temperature for 24 h.

### Bracket debonding

#### Universal testing machine

**Fig. 2:**
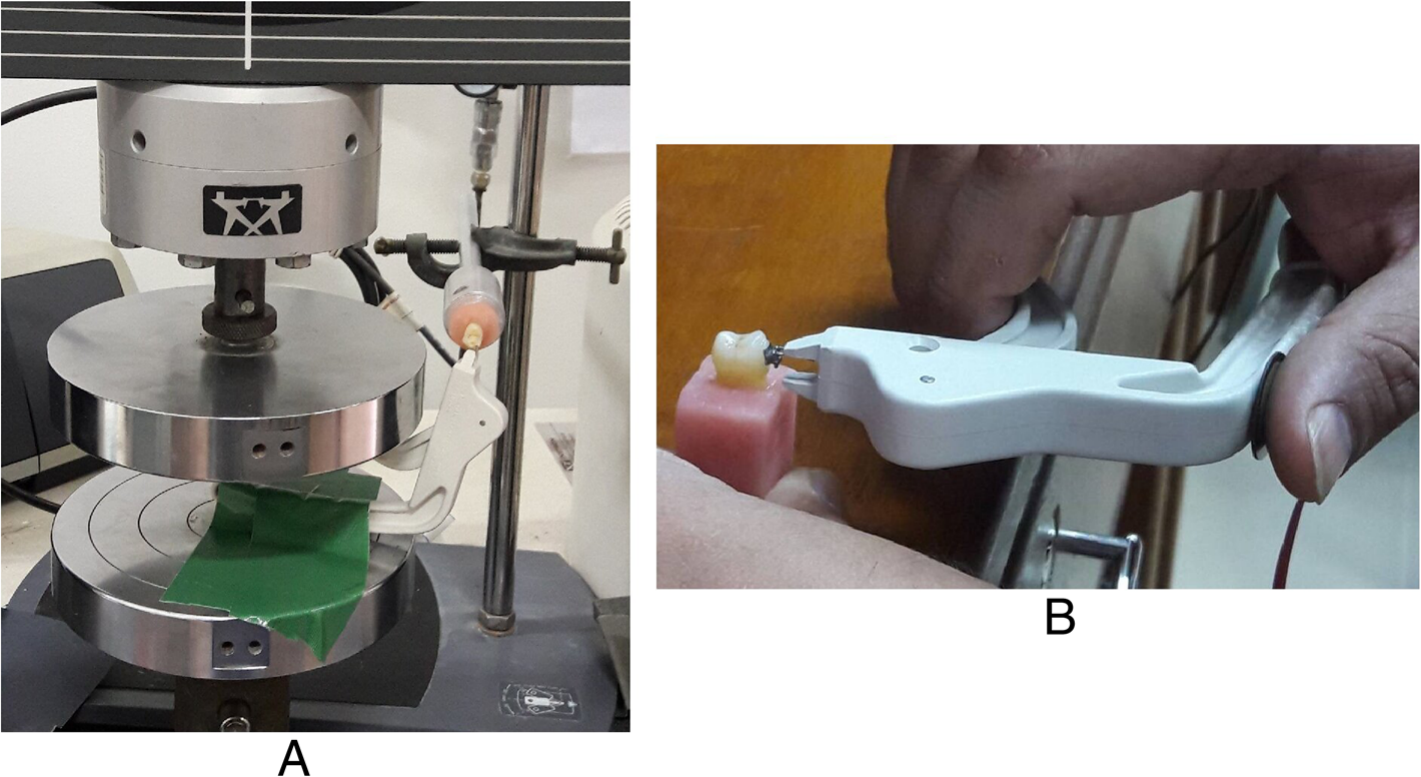
Validation test. **a** LODI simulated bracket debonding. **b** Bracket debonding by the prototype device

To simulate the debonding mechanism of the LODI, the instrument itself was mounted on the universal testing machine (Model-3366, INSTRON, USA) and fixed in position by a heavy-duty adhesive cloth tape (SB Tape, Selangor, Malaysia) (Fig. 2a). The samples were inserted into that metallic hollow cylinder held with a stand. The brackets were facing downwards towards the bracket-fixing wire loop of the LODI. The wire loop of the LODI was engaged on one of the bracket wings, and the compression force was applied on the plier handles by the load cell of 10 kN and at a crosshead speed 5 mm/min [16]. The maximum debonding forces were recorded in newton (N) units by the associated software (INSTRON Series IX/s Software, version 8.25.00, USA).

#### Prototype device

Orthodontic brackets from the thirty (30) samples were debonded by a single clinician for the validation study. For the reliability study, thirty-nine (39) samples were randomly and equally divided into three (3) groups. For intra-examiner reliability, groups 1 and 3 were debonded by the same clinician. Brackets in group 2 were debonded by another calibrated clinician and compared with group 1 for inter-examiner reliability. In all cases of bracket debonding, the device was held in a standardized position. The device was held with the thumb on the force sensor and the rest of the fingers on the anterior arm. After engaging on one of the bracket wings by the wire loop, the force was applied with the thumb on the sensor to compress the plier handles until debonding (Fig. 2b). The maximum sensor output at the incidence of bracket debonding was noted and converted into force (in newton units) by the regression equation.

Following bracket debonding by the prototype device, the enamel surface of the specimen was inspected under the stereomicroscope (Celestron, CA, USA) at × 30 magnification to evaluate the bracket failure pattern by the 4-point scale Adhesive Remnant Index (ARI): score 0 = no adhesive left on the tooth, score 1 = less than half of the adhesive left on the tooth, score 2 = more than half of the adhesive left on the tooth, and score 3 = all the adhesive left on the tooth, with distinct impression of the bracket base [17].

### Statistical analysis

The normality of the data distribution was confirmed by the one-sample Kolmogorov-Smirnov test. Independent sample *t* test was applied to detect the mean difference of orthodontic bracket debonding force between the universal testing machine and the prototype device. Intraclass correlation coefficient (ICC) test with a 95% confidence interval was utilized to evaluate the reliability of the prototype device. ICC model (3, 1) was applied for the intra-examiner reliability and ICC (2, 1) for the inter-examiner reliability. The level of significance was considered *p* < 0.05.

### Ethical approval

The study was ethically approved by the human research and ethics committee (study protocol code: USM/JEPEM/17020075).

## Results

### Validation study

No significant difference (*p* = 0.072) of mean orthodontic bracket debonding force was noted between the groups measured by the universal testing machine and the prototype device (Table 1). The mean debonding force measured by the universal testing machine and the prototype device was 10.43 ± 2.71 N and 9.36 ± 1.65 N respectively.

**Table 1:** Comparative debonding force values between the universal testing machine and the prototype device

Variables	Mean ± standard deviation		*T* statistics	*p* value
	Universal testing machine (*n* = 30)	Prototype device (*n* = 30)		
Debonding force (N)	10.43 ± 2.71	9.36 ± 1.65	1.837	0.072

*n*, number of samples; *N*, newton units of force

*p* < 0.05

### Reliability study

ICC models (3, 1) for intra-examiner and (2, 1) for inter-examiner reliability were applied. The ICC value for intra-examiner and inter-examiner reliability were 0.942 and 0.921 respectively with a 95% confidence interval (Tables 2 and 3).

**Table 2:** Intra-examiner reliability of the prototype device

Variable	Measures	Intraclass correlation	95% confidence interval		*F* test with true value 0			
			Lower bound	Upper bound	Value	df1	df2	*p* value
Debonding force	Single	0.942	0.786	0.983	42.642	12	12	< 0.001

**Table 3:** Inter-examiner reliability of the prototype device

Variable	Measures	Intraclass correlation	95% confidence interval		*F* test with true value 0			
			Lower bound	Upper bound	Value	df1	df2	*p* value
Debonding force	Single	0.921	0.764	0.975	22.874	12	12	< 0.001

## Discussion

This is a novel method as previously no study was found in the literature that measured orthodontic bracket debonding force utilizing FSR. In dynamic measurements, FSRs exhibited higher accuracy exceeding 95% [18]. Previously in dentistry, FSR was able to measure bite force with 93% reliability and similarly to widely accepted strain gauge equipped bite fork [19]. The FSR was attached on the manufacturer-made LODI without any structural modification of the plier. Manufacture-made pliers apply force in a precise direction and therefore are capable of debonding brackets consistently at the bracket-adhesive interface, limiting the enamel damage [20]. From the patients’ perspective, LODI was considered the least discomforting among other debonding methods [21, 22]. Also, brackets were found to be less distorted and considered for recycling when debonded by the LODI [23].

In this study, peak debonding forces in newton (N) units but not the conventional average stress in megapascal (MPa) units were reported as results. Because according to the finite element analysis if the peak force at the site of application is responsible for the entire bracket debonding, then the average stress which is calculated dividing the peak force by the entire bracket surface area does not accurately reflect the debonding process [7]. Many studies may report bond strength of orthodontic brackets in average stress in order to compare the results with other studies. Such comparisons of average stress results from different testing protocols are invalid as the results are incompatible [1].

Validation of the prototype device is crucial to justify its clinical application. For validation, the comparison between the “gold standard” universal testing machine and the prototype device was made as some of the previous prototype devices [4, 6, 12, 13]. One study reported a difference of orthodontic bond strength between the universal testing machine and their prototype bracket debonding device due to the difference in the type of force application and rate of loading [6]. The universal testing machine is designed to apply either true shear or tensile force, and in contrast, the debonding devices apply a combination of shear/peel, tensile, or torsional loading modes in all directions [7]. As such comparisons for validation remain doubtful, the present study mounted the debonding plier (LODI) itself on the universal testing machine to simulate the clinical debonding while measuring the orthodontic bracket debonding force. As a result, in both instances, a similar method of bracket debonding was followed which justifies the comparison of the mean debonding force. In both cases, compressive forces were applied to the plier handles and the bracket was debonded according to the manufacturer’s standardized direction. This idea of simulated bracket debonding by mounting the debonding plier on the universal testing machine was obtained from the previous in vitro studies [24–26]. In a study, LODI-simulated bracket debonding was performed by engaging the fishing line wire on to one of the bracket wings instead of using the LODI itself and the mean debonding force (32.5 ± 4.9 N) was higher with greater standard deviation in comparison to the current study (10.43 ± 2.71 N) [27]. To simulate LODI mechanism, the wire may be engaged to one bracket wing, but it exerted true tensile force by the universal tensile machine. Another study mounted LODI on the universal testing machine which resulted in a mean debonding force of 6.8 ± 1.2 N [26]. Despite the similar method, a small difference of mean bracket debonding force may be attributed to the use of different brackets, bonding techniques, and adhesives. The current study resulted in no significant difference (*p* = 0.072) of mean orthodontic bracket debonding force between the universal testing machine and the prototype device (Table 1) which confirms that this prototype device can be a useful tool to measure orthodontic bracket debonding force.

One of the limitations of this prototype is that it has to be held in a standardized fixed position all the time to perform bracket debonding. As the force sensor is human touch sensitive, bracket debonding without pressing the sensor will result in an error. For this reason, the device had to be held in all cases by placing a thumb on the active area of the force sensor which was attached to the posterior arm of the LODI handle and the rest of the fingers on the anterior arm. Thereby, the entire force was applied by the thumb on the sensor that compressed the LODI handles until bracket debonding. To confirm the consistency of such a modified device holding position while debonding orthodontic brackets, both intra- and inter-examiner reliability tests were required. For standardization of the samples, same brackets were bonded on the similar tooth surface by a single clinician using the same adhesive, surface preparation, and bonding technique. Intraclass correlation coefficient (ICC) value for intra- and inter-examiner reliability was respectively 0.942 and 0.921 indicating excellent reliability [28].

An ideal debonding force should be able to remove brackets with least damage to the underlying enamel. It has been reported in a study that orthodontic bond strength should be less than 9.7 MPa to prevent enamel damage [29]. Another study confirmed a clinically acceptable bond strength to be 5.9–7.8 MPa [30]. Although it is invalid to compare as the present study did not report the results in average stress of MPa units, it can be estimated that the mean debonding forces obtained in this study are much lower than these established values. This can be explained by the variation in the mode and location of force application along with the testing device. The prototype used in this study principally applies tensile force with shear-peel and torsional components in comparison to either true shear force or tensile force exerted by the universal testing machine. In comparison to traditional laboratory tensile debonding test, LODI (i.e., the plier used in the current study) required a lower debonding force [27]. According to the finite element analysis, this lower debonding force is due to the distribution of higher asymmetric stress within the structures of the bracket-adhesive system [31]. For the comfort of the subject, 1000 g of force which is equivalent to 9.8 N was considered as an appropriate force limit to be applied directly to the tooth [32]. In the current study, the mean debonding force measured by the prototype device was within this value.

**Fig. 3:**
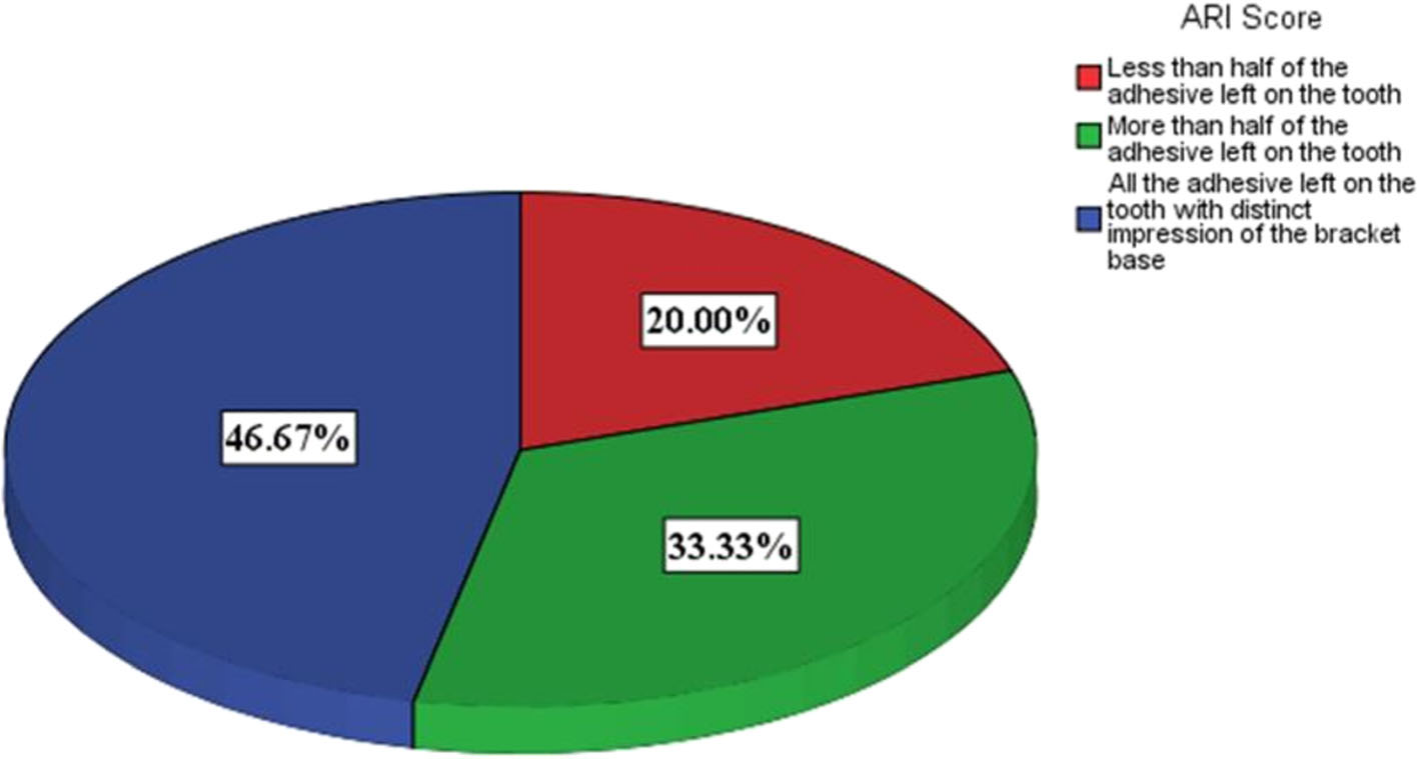
Bracket failure pattern by the prototype device in 4-point ARI

ARI is the most common tool for the subjective and qualitative analysis of the orthodontic bracket failure in order to assess the enamel damage. In this study, ARI score 3 was most predominant (Fig. 3) which means bracket failure by the prototype mostly occurred at the bracket-adhesive interface and thus leaving the underlying enamel intact [33, 34].

## Conclusion

The prototype orthodontic bracket debonding device utilizing FSR introduced in the current study can be considered useful to measure clinical bonding efficiency of a wide range of orthodontic brackets, adhesives, surface preparation, and bonding techniques that are evolving regularly for the uninterrupted and better treatment outcome. Despite the little modification in gripping, the device was proved to be accurate and reliable and conserved the underlying surface enamel.

## Data Availability

All data available within the article in the form of tables, result text, and figures.
